# Extracellular trap can be trained as a memory response

**DOI:** 10.1080/21505594.2022.2046950

**Published:** 2022-03-07

**Authors:** Yu Gao, Jian-Gang Zhang, Zhen-Zhen Liu, Ke Ma, Xiao-Qi Lin, Jia-Bao Zhang, Wei Chen, Yong-Jun Yang

**Affiliations:** Key Laboratory of Zoonosis Research, Ministry of Education, College of Veterinary Medicine, Jilin University, Changchun, China

**Keywords:** Extracellular trap, trained immunity, trained memory, innate immune, memory response, antimicrobial

## Abstract

Extracellular trap (ET) appears as a double-edged sword for the host since it participates in host immune defense by entrapping pathogens, while excessive ET release also contributes to various diseases progression including atherosclerosis, cancer, and autoimmune disorders. A better understanding of ET formation and regulation will be beneficial for developing strategies for infection control and ET-associated disease treatment. There is some evidence indicating that prior infection can enhance extracellular killing. Neutrophils from cancer or sepsis are predisposed to generate ET. It is reasonable to suspect that ET may be trained to form as a memory response, just like cytokine memory response termed “trained immunity.” The mice were intraperitoneally injected with heat-killed *Candida albicans* (HK-*C. albicans*), 3 days later bone marrow-derived macrophages (BMDM) were isolated and challenged with *Clostridium perfringens* as a second stimulation. We found that HK-*C. albicans* priming enhanced ET formation upon *Clostridium perfringens* infection, accompanied by increased extracellular killing capacity. Mannan priming also enhanced ET formation. Since ETs memory was induced in chicken PBMC, ETs memory may be evolutionarily conserved. Moreover, mTOR was required for ETs memory response. Collectively, this study showed that ETs can be trained as a memory response and indicated that memory property of ETs should be considered during the understanding of recurrent infection and ET-associated disorders.

## Introduction

Extracellular trap (ET) is composed of chromosomal DNA decorated with characteristic granule proteins, such as histones, myeloperoxidase (MPO), and neutrophil elastase (NE) [1], which is released from activated immune cells exposed to stimuli including bacteria, viruses, or fungi and inflammatory mediators [2–4]. The macrophage ETs (METs) are similar to neutrophil ETs (NETs) [1,2]. A critical signaling event in ETs is the decondensation of chromatin, which is facilitated by peptidylarginine deiminase 4 (PAD4). PAD4 is a histone-modifying enzyme, which converts histone arginine (Arg) or monomethyl-Arg to citrulline (Cit) [5,6]. ETs have been discovered as a protective defense mechanism against various pathogens [7–11], and subsequently, dysregulation of ETs has been linked to various human autoimmune and inflammatory diseases including atherosclerosis, autoimmune diseases, acute injuries, asthma [12–16]. A recent study confirms that colon cancer cells enhanced the level of METs and METs promoted the invasion of colon cancer cells [3].

Several studies on increased NET formation due to previous infection or disease attracted our attention. Treatment of neutrophils from mammary tumor-bearing mice with LPS showed a significant increase in NET formation and citrullinated histone H3 expression compared to tumor-free mice, suggesting that a low dose infection leads to an increased NET formation in a tumor-bearing host [4]. Neutrophils from chronic myelogenous leukemia (CML), breast, and lung cancer are predisposed to generate NET, the leading cause of cancer-associated thrombosis [4]. Similarly, NETs released into the vasculature have the capacity for bacterial trapping during sepsis, and prevent dissemination [5,6]. In another study, neutrophils isolated from surviving patients with sepsis showed an increased NET formation in response to subsequent phorbol 12-myristate 13-acetate (PMA) stimulation compared with neutrophils from healthy donors [7]. Neutrophils isolated from type 1 and type 2 diabetic humans and mice were primed to produce NETs [8], associated with higher PAD4 and CitH3 expression. Since the NET from patients with T2D results in impaired NETs killing activity, patients are more susceptible to hypervirulent *K. pneumoniae* infection [9].

Thus, there is growing evidence indicating that priming signals such as pathogens and diseases activate the memory of extracellular traps (ETs) and potently increase ETs release in response to a second stimulation. The positive or enhanced response has been coined trained immunity (also known as innate immune memory), which can produce an enhanced immune response against secondary infection of related or unrelated pathogens [10,11]. The resulting immune enhancement is manifested as the enhanced ability of phagocytosis and increased production of cytokines [12].

*Clostridium perfringens* causes an opportunistic infection among humans and livestock, which restricts the development of animal husbandry [13]. *Clostridium perfringens* produces more than 16 different toxins, leading to the virulence of different *C. perfringens* strains [14]. In this study, we aimed to investigate the effect of priming signals on extracellular trap formation, revealing the memory response of ETs. Our results showed that priming with heat-killed *C. albicans* (HK-*C. albicans*) contributes to decreasing the extracellular *C. perfringens* burden and prevents reinfection. Further results supported the priming signal enhanced the release of extracellular traps after secondary stimulation with heterogeneous pathogens. This work suggested the trained memory of ETs could provide more effective prevention and treatment of microbial infection, and possibly a mechanism of ET-associated diseases, which is a potential target for the treatment of various diseases.

## Materials and methods

### Animals

C57BL/6J mice were obtained from Jackson Laboratories (Bar Harbor, ME, USA). Experiments were conducted with 6- to 8-week-old-age matched mice housed in plastic cages, provided water ad libitum, and fed sterilized laboratory chow.

HY-LINE VARIETY BROWN chickens were used at 3–5 days, water ad libitum and a stipulated amount of commercial feed daily were provided.

All animal studies were approved by the Animal Welfare and Research Ethics Committee at Jilin University (No. 20,150,601).

### Preparation of heat-killed *Candida albicans*

*Candida albicans* BNCC337321 (BeNa Culture Collection) were grown in YM medium at 37°C for 8 h, suspended in a PBS solution, washed three times, and killed at 100 degrees for 1 h.

### *Clostridium perfringens* culture

*Clostridium perfringens* ATCC13124 (American-type culture collection, ATCC) were grown in the brain heart infusion broth (BHI) medium at 37°C anaerobic for 8 h.

### In vivo animals treatments

Priming of innate immune cells was performed in animals by IP injection of HK-*C. albicans* or mannan (1 mg; Solarbio M870) in a volume of 100 μL per mouse or chicken. The non-primed group was injected with the same volume of PBS. HK-*C. albicans* or mannan was administered 3 days before sacrifice.

In inhibitor experiments, mice were injected IP with rapamycin (Selleck; AY -22,989) 30 min prior to HK-*C. albicans* injection. Rapamycin stock was solubilized in DMSO at a concentration of 50 mg/mL and further diluted in PBS. Mice were injected IP with 4 mg/kg rapamycin 100 μL of PBS containing 2% DMSO. Control mice were injected with PBS containing 2% DMSO. Three days after the HK-*C. albicans* treatment, mice were sacrificed.

### Isolation of bone-marrow-derived macrophages

Bone-marrow-derived macrophages were obtained by flushing mouse femur and tibia bone marrow. Cells were cultured with RPMI 1640 medium (Gibco; 31,800–002) containing 25% L929-cell conditioned medium, 10% FBS, 100 U/mL ampicillin, and 100 μg/mL streptomycin to promote cell differentiation in an incubator at 37°C and 5% CO_2_. The differentiated macrophages were collected and were resuspended in RPMI 1640 medium containing 10% FBS, 100 U/mL ampicillin, and 100 μg/mL streptomycin.

### Isolation of peripheral blood monocytes

Peripheral blood monocytes were obtained by collecting chicken blood through jugular vein blood, using a chicken peripheral blood mononuclear cell separation kit (Solarbio P5250), obtained white mononuclear cell layer by density gradient centrifugation. Cells were suspended and adjusted to 6 × 10^6^ cells/mL using RPMI 1640 medium containing 10% FBS, 100 U/mL ampicillin, and 100 μg/mL streptomycin. Then, cells were incubated for 4 h in an incubator at 37°C and 5% CO_2_ and washed out into non-adherent cells with warm PBS to select adherent monocytes.

### Extracellular bacterial killing assay

Primed and non-primed cells were re-challenged with *C. perfringens* at a multiplicity of infection (MOI) of 5 for 3 h. The samples of culture supernatants were collected, diluted into sterile isotonic saline, plated on BHI agar plates, and incubated overnight at 37°C anaerobic for bacterial enumeration.

### Quantification of ETs release

As described previously [15], for quantitative detection of extracellular DNA, primed and non-primed cells were stimulated with *Clostridium perfringens* (MOI = 5) at different time points (2 h, 3 h, and 4 h) and centrifuged at 3000 rpm for 5 min to collect the supernatant. An equal amount of RPMI 1640 medium was added to the uninfected control group. SYTOX Green (5 μM, Invitrogen; S7020) was added to the supernatants of the cells to detect extracellular DNA. Read the fluorescence value in a fluorescence microplate reader. The excitation and emission wavelengths were 485 and 530 nm, respectively. For the trained experiments, the final results ratio of the *C. perfringens* infected group to the uninfected group is shown. Cells without *C. perfringens* were assumed to be 1. For inhibitor experiments, non-primed BMDMs infected without *C. perfringens* were used as control. DNA release was quantified and presented as ratio vs control.

### Western blotting

BMDMs were harvested at designated time points after *C. perfringens* infected (MOI = 5). As a control, to an identical cell culture, the same volume of PBS alone was added to the RPMI 1640 medium. Post infection, cells were homogenized in a lysis solution supplemented with a complete protease inhibitor cocktail (Sigma-Aldrich; P8340). The samples were then centrifuged at 13,000 rpm for 10 min, and supernatants were collected and used for western blotting analysis. The samples were separated by 12% SDS-PAGE gels and transferred to a polyvinylidene fluoride membrane. After a block with 5% skim milk, the membranes were incubated with primary antibodies against CitH3 (Abcam ab5103), PAD4 (Proteintech 17,373-1-AP), and GAPDH (Proteintech 10,494-1-AP).

### Cytokine production

To measure the production of cytokine, BMDMs were stimulated with *Clostridium perfringens* (MOI = 5). An equal amount of PBS was added to the uninfected group. After 1 h, cells were treated with RPMI 1640 medium containing 30 μg/mL gentamicin, 200 U/mL penicillin, and 200 μg/mL streptomycin to kill extracellular bacteria. After 1 h, discard the culture medium and replace it with RPMI 1640 culture medium containing 10 μg/mL gentamicin and 100 U/mL penicillin, and 100 μg/mL streptomycin to continue culturing for 22 h. The cell supernatants were collected and used for inflammatory cytokine examination with TNF-α ELISA kits following the R&D Systems (DY410) instruction.

### LDH assays

The cytotoxicity was tested using the lactate dehydrogenase assay. Collect the cell culture supernatant at 3-h post-infection with *Clostridium perfringens* (MOI = 5) as described above. At the same time, untreated cell wells were repeatedly frozen and thawed three times, and then the supernatant was collected as a positive control. The Cytotoxicity Assay (Promega G1781) was used to detect the content of lactate dehydrogenase. Read the absorbance at 490 nm. In the final data processing, the ratio of each group to its positive control absorbance value was plotted.

### NO production

The generation of nitric oxide (NO) was measured by the Griess reaction. To better measure the production of NO, cells were stimulated with *Clostridium perfringens* (MOI = 10). An equal amount of PBS was added to the uninfected group. After 1 h, cells were treated with RPMI 1640 medium containing 30 μg/mL gentamicin, 200 U/mL penicillin, and 200 μg/mL streptomycin to kill extracellular bacteria. After 1 h, discard the culture medium and replace it with RPMI 1640 culture medium containing 10 μg/mL gentamicin and 100 U/mL penicillin, and 100 μg/mL streptomycin to continue culturing for 22 h. The culture supernatants were collected, and NO production was measured according to the indication on the NO assay kit (Beyotime S0021S).

### Statistical analysis

The data were expressed as mean ± SEM. All experiments were independently performed three times. Data sets with two independent groups were analyzed for statistical significance using unpaired Student’s t-test. All p values less than .05 were considered significant (*p < .05, **p < .01, and **p < .001). NS represents that there is no statistical difference. GraphPad Software (GraphPad Software, La Jolla, CA, USA) performed the statistical analysis.

## Results

### Treatment of mice with HK-*C.* *albicans* shows increased antibacterial functions ex vivo

**Figure 1.**
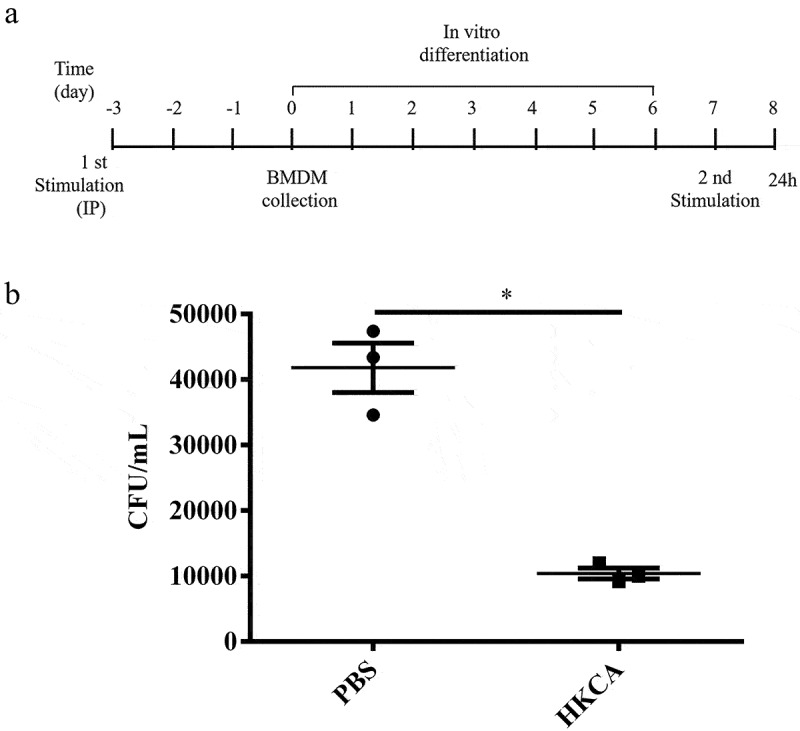
HK-*C.* *albicans* primed BMDMs show increased antibacterial functions. a) Chronogram of the BMDM experiment. C57BL/6 WT mice were injected IP with an inducer of trained immunity in 100 μL before bone marrow cells were collected. Control mice were injected IP with the same volume PBS. Bone marrow cells were differentiated into bone marrow-derived macrophages (BMDMs) for six days, plated, and stimulated with *Clostridium perfringens* at a MOI of 5 or PBS vehicle. b) the killing ability of BMDM primed with PBS or HKCA (HK-*C.* *albicans*). 3 h post *C.* *perfringens* (MOI = 5) infection, viable bacteria were plated on BHI agar to determine CFU counts.

*Candida albicans* fulfills its role as a well-known inducer of trained immunity that relies on Dectin-1. The receptor of Dectin-1 senses *C. albicans* invasion by recognizing β-glucan, then activates downstream inflammation signaling pathways and induces an increase in pro-inflammatory cytokines such as TNF-α, IL-6 [16]. It has been reported that the heat killing of *C. albicans* contributes to the surface exposure of β-glucan [17]. We established a mice-trained immunity model using intraperitoneal injection with heat-killed *Candida albicans* (1 × 10^6^ CFU). Three days later, bone marrow was harvested and differentiated into bone-marrow-derived macrophages (BMDMs). Then, macrophages were stimulated with *Clostridium perfringens* (*C. perfringens*) in vitro (Figure 1(a)). Consistent with the previous report [18], TNF-α production was measured after stimulation with *C. perfringens* for 24 h. Without *C. perfringens* challenging, TNF-α were low or undetectable in both *C. albicans*-primed and non-primed cells (data not shown). However, *C. albicans*-primed BMDM released more TNF-α than non-primed BMDM upon *C. perfringens* challenging (Figure S1). To investigate whether priming with HK-*C. albicans* increased the killing capacity against *C. perfringens*, and next we determined the killing ability of BMDM by counting extracellular bacteria. Importantly, priming with HK-*C. albicans* prior to *C. perfringens* challenge significantly enhanced the ability of BMDM to kill extracellular bacteria (Figure 1(b)).

Data are shown as the mean ± SEM. n = 3 per group. Data from are three independent experiments. Statistical significance is indicated by *p < 0.05.

The enhanced immune response of BMDMs due to prior interactions with HK-*C. albicans* stimuli are suggested for trained immunity. Further, as expected, priming with HK-*C. albicans* induced the memory response of the ability to kill *Clostridium perfringens*.

### Treatment of mice with HK-*C.* *albicans* leads to a memory response of ET ex vivo

**Figure 2.**
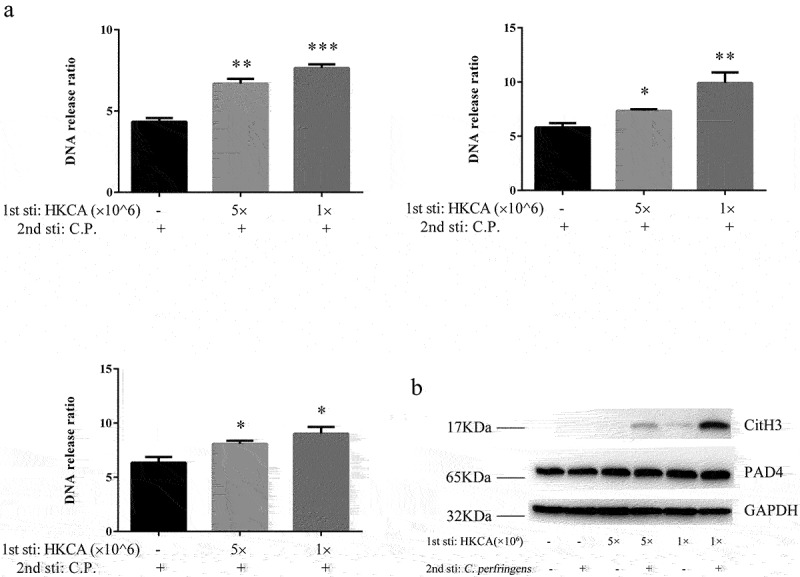
Trained BMDMs increased release of extracellular traps. a) Extracellular DNA release from BMDM cultures was quantified. The release of extracellular DNA from BMDM (primed with PBS, 1 × 10^6^ CFU and 5 × 10^6^ CFU heat-killed *C.* *albicans*), was detected by SYTOX Green 2 h,3 h,4 h post-stimulation with the *C.* *perfringens* (MOI = 5). Extracellular DNA from cells was incubated without *C.* *perfringens* assumed as 1. Results are shown as fold increase relative to cells without *C.* *perfringens*. b) Western blot analysis of protein expression. Non-primed and HK-*C.* *albicans*-primed BMDMs were infected with *C.* *perfringens* (MOI = 5) or not for 3 h, whole cell lysates were analyzed for CitH3, PAD4 and GAPDH by Western blotting.

Macrophages generated extracellular traps are involved in the elimination of pathogens similar to neutrophils [2,15]. Since ET has been associated with extracellular pathogen killing, we investigated whether the enhanced extracellular bacterial killing was mediated by the release of ETs. For this purpose, the extracellular DNA release in primed and non-primed BMDMs was quantified by SYTOX Green. This DNA-intercalating dye is impermeant to live cells as a quantitative analyzing method of ETs formation. Experiments were performed as described above. Both high and low doses of HK-*C. albicans* induced a significantly higher level of DNA release in response to *C. perfringens* than PBS priming. BMDM primed with 1 × 10^6^ CFU heat-killed *C. albicans* showed a more effective ability for DNA release compared to 5 × 10^6^ CFU (Figure 2(a)). However, the DNA release of BMDMs was at the basal level without *C. perfringens* stimulation, and there was no statistical difference (data not shown). Peptidylarginine deiminase 4 is the essential enzyme of ET formation, locates at the nuclear, and catalyzes histone citrullination [19,20]. Citrullinated histone H3 has previously been shown to be a marker for NET. The expression of CitH3 at 3 h post-stimulation was significantly higher in HK-*C. albicans* primed BMDM. By contrast, 1 × 10^6^ CFU heat-killed *C. albicans* priming showed the highest amounts of CitH3. Consistent with CitH3 expressions, priming with HK-*C. albicans* also upregulated the expression of PAD4 (Figure 2(b)). The cytotoxicity was evaluated by detecting the lactate dehydrogenase (LDH) content, showing no difference in LDH level between the primed and non-primed BMDM (Figure S2), suggesting similar macrophage health.

Data are shown as the mean ± SEM. n = 3 per group. Data from are three independent experiments. Statistical significance is indicated by *p < 0.05, **p < 0.01, and ***p < 0.001.

Altogether, these data suggest that HK-*C. albicans* priming enhanced the ability to kill extracellular bacteria is mediated by ET formation. The above results indicate that pre-treatment with HK-*C. albicans* leads to the memory response of ETs, which is consistent with cytokine production in trained immunity.

### The mannan derived from Saccharomyces cerevisiae is involved in ET memory response

**Figure 3.**
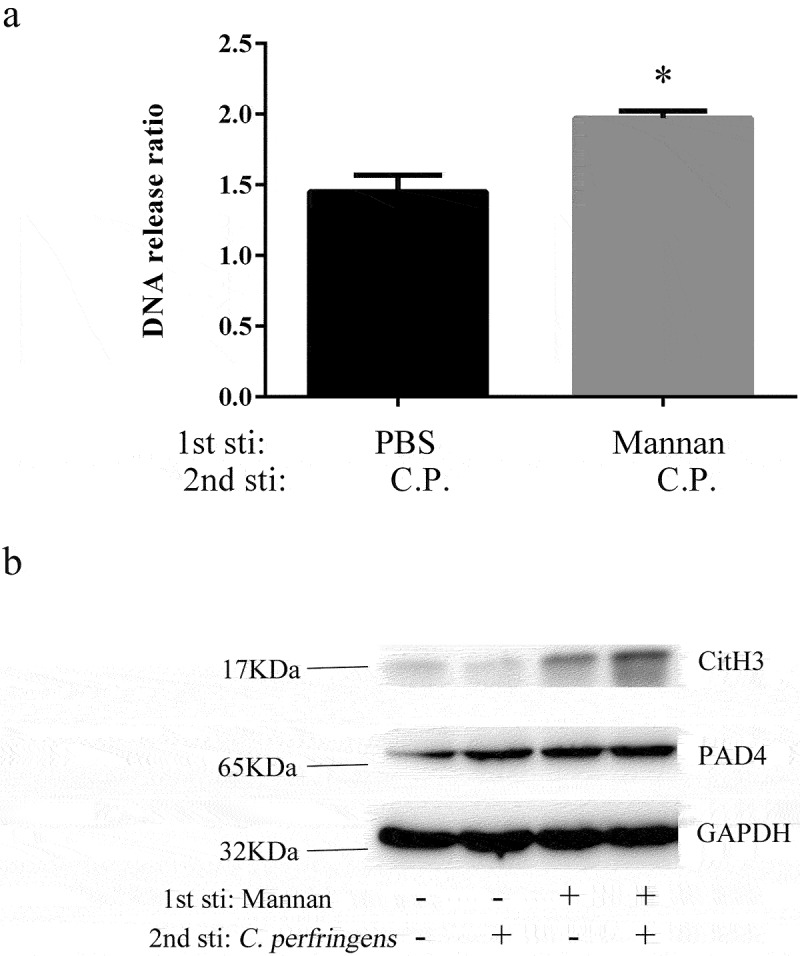
Mannan-Induced BMDM enhanced the memory response of ETs. a) Extracellular DNA release from BMDM cultures was quantified. BMDMs from non-primed and mannan-primed (1 mg/ml) mice were infected with *C.* *perfringens* (MOI = 5) or not. 3 h post-infection, the extracellular DNA in cell supernatants was stained with SYTOX Green and detected with a fluorescent reader. BMDMs incubated without *C* *perfringens* were used as control, assumed 1. Results are shown as fold increase relative to cells without *C.* *perfringens*. b) Western blot analysis of protein expression. Non-primed and mannan-primed BMDMs were infected with *C.* *perfringens* (MOI = 5) or not for 3 h, whole cell lysates were analyzed for CitH3, PAD4 and GAPDH by Western blotting.

β-glucan extracted from *Candida albicans* and *Saccharomyces cerevisiae* (*S. cerevisiae*) results in a heightened innate immune response upon exposure to secondary infections by activating the Dectin-1 receptor on particular immune cells [21]. Similar to β-glucan, the mannan derived from the yeast cell wall could stimulate both innate and acquired immunity [22]. The previous study showed that cells primed with mannan extracted from *Candida albicans* did not increase the production of the pro-inflammatory cytokines [16]. To determine whether mannan triggers the memory response of extracellular traps, mice were injected with mannan, which was derived from *S. cerevisiae*, and then mice were sacrificed to collect BMDM for *ex vivo* stimulation with *C. perfringens*. Experiments were performed as described above. Three hours after PBS or *C. perfringens* infection, extracellular DNA release was determined. There was no statistical difference between baseline conditions, which were cultured without bacteria. We found a 13% increase in extracellular DNA release from mannan-primed BMDM infected with *C. perfringens* compared with the non-primed BMDM (Figure 3(a)). Then, we found an increased expression of CitH3 in mannan-primed BMDM infected with *C. perfringens* by western blot analysis (Figure 3(b)). It suggests that mannan derived from *Saccharomyces cerevisiae* priming can induce the memory response of ETs.

Data are shown as the mean ± SEM. n = 3 per group. Data are pooled from 2 independent experiments. Statistical significance is indicated by *p < 0.05.

### ETs released by chicken peripheral blood mononuclear cells is trained as a memory response

**Figure 4.**
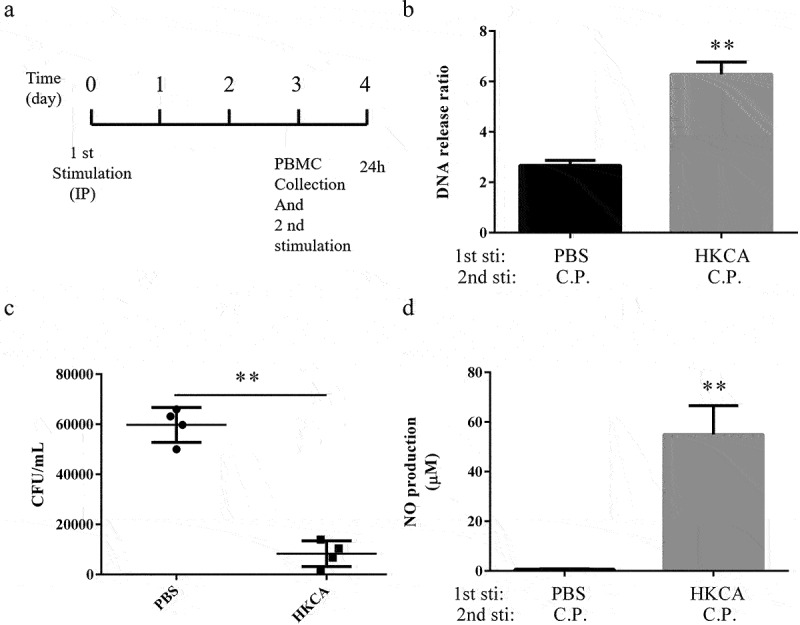
HK-*C.* *albicans* leads to trained memory of ETs in chicken PBMC. a) Chronogram of the chicken PBMC experiment. the chickens have been injected IP with 1 × 10^6^ CFU heat-killed *C.* *albicans* in 100 μL before the collection of PBMCs. Adherent cells were second stimulated with *Clostridium perfringens* or PBS vehicle at the designed time points. b) the release of extracellular DNA from HK-*C.* *albicans*-primed PBMCs was detected by SYTOX Green 3 h post-stimulation with the *Clostridium perfringens* (MOI = 5) or not. PBMCs incubated without *C* *perfringens* were used as control, assumed 1. Results are shown as fold increase relative to cells without *C.* *perfringens*. c) the killing ability of PBMCs. The extracellular bacteria of PBMC 3 h post *C.* *perfringens* (MOI = 5) infection was assessed by plated on a BHI agar plate. d) PBMCs nitric oxide (NO) production. Non-primed and HK-*C.* *albicans* primed PBMCs infected with *C.* *perfringens* (MOI = 10), 24 h post-infection, the cell culture supernatants were collected and nitric oxide was measured by Griess reaction. PBMCs incubated without *C* *perfringens* were used as a control (data not shown).

Given that induction of trained immunity in multiple species could be achieved via a priming event [23–26], to explore whether the memory response of ETs is evolutionarily conserved in various species, we next considered the trained memory of ETs in poultry. In these experiments, chickens were primed as in the previous model. Chickens received an IP injection of either PBS or HK-*C. albicans* (1 × 10^6^ CFU), at 3-day post-HK-*C. albicans* or -PBS injection, we collected peripheral blood mononuclear cells (PBMC) and re-challenged with *C. perfringens* (Figure 4(a)). Avian granulocytes (heterophils) form ETs that can limit and further kill pathogens [27]. Although monocytes incubated in RPMI 1640 medium without any bacteria had no statistical difference in DNA release, which was at the basal level. Re-challenge with *C. perfringens* (MOI = 5) resulted in a significantly higher release of extracellular DNA in HK-*C. albicans*-primed PBMC (Figure 4(b)), thereby it showed increased the ability to kill bacteria of PBMC (Figure 4(c)). Consistent with previous reports about trained immunity in chicken [28], we observed an increased production of antimicrobial NO upon stimulation with *C. perfringens* (MOI = 10) is stronger in HK-*C. albicans*-primed PBMCs compared to non-primed PBMCs (Figure 4(d)). Unstimulated cells were used as controls for the basal level. However, we did not find any NO production in PBMCs upon stimulation with *C. perfringens* at an MOI of 5 (Data not shown).

Data are shown as the mean ± SEM. n = 3 per group. Data from are three independent experiments. Statistical significance is indicated by **p < 0.01.

Altogether, measurement of NO, as well as measurement of ETs, showed heightened innate immune functions and the memory response of ETs in chicken PBMCs. It suggests that the memory response of ETs not just in mammals but also in poultry, which is evolutionarily conserved in various species.

### The mTOR is required to memory response of extracellular trap

**Figure 5.**
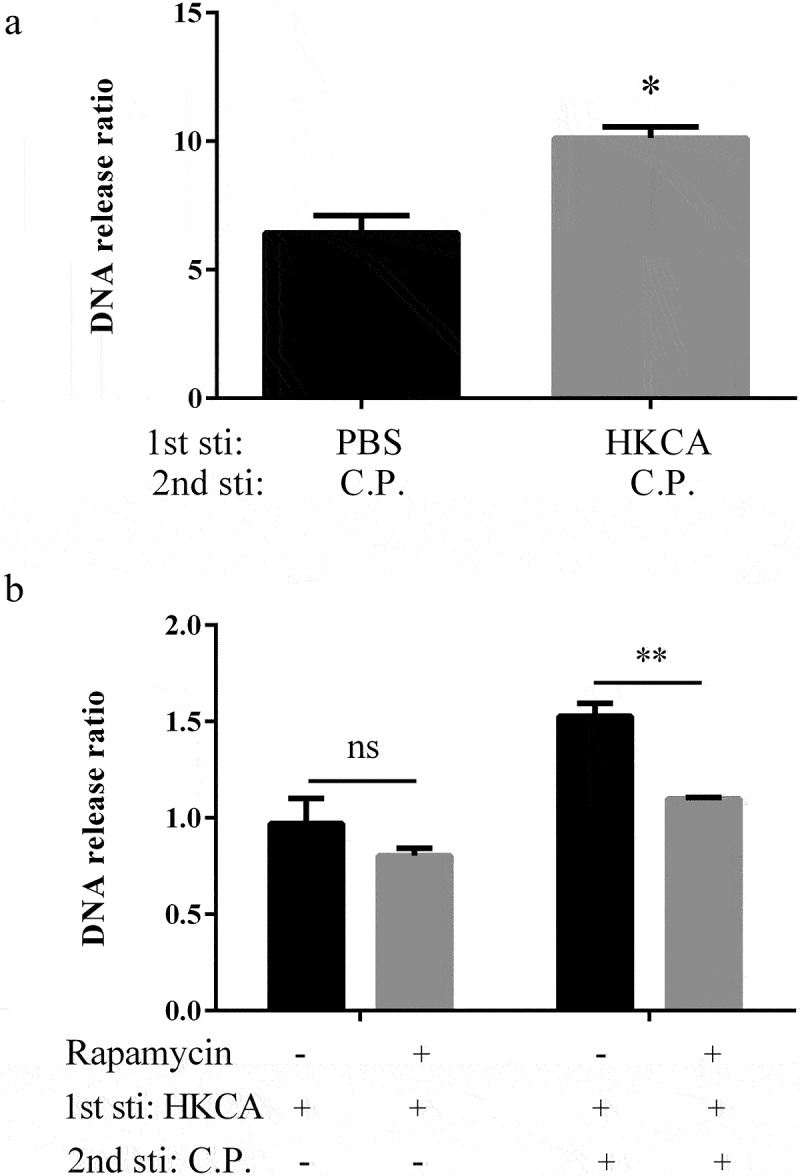
Rapamycin inhibits the trained memory of ETs. Intraperitoneal injection in C57BL/6 WT mice with 4 mg/kg rapamycin for 30 min prior to HK-*C.* *albicans* (1 × 10^6^ CFU) primed. Animals were sacrificed 3 days after injection and collected BMDMs. BMDMs infected with *C.* *perfringens* (MOI = 5) or PBS vehicle and fluorescence of extracellular DNA was measured 3 hours after infection. a) Extracellular DNA release was quantified. BMDMs stimulated with PBS were used as control. Fold changes in DNA release obtained in BMDM after stimulation with *C.* *perfringens*. the ratio of DNA release by *C.* *perfringens* versus PBS stimulation BMDMs is presented. b) Non-primed BMDMs infected without *C.* *perfringens* was used as control, was assumed to be 1. DNA release was quantified and presented as ratio vs control.

C.*albicans* induced monocytes metabolic shift, including aerobic glycolysis (the “Warburg effect”), increased glucose consumption and lactate production, which depended on the Dectin-1-Akt-mTOR-HIF-1α pathway [29]. Intraperitoneal injection in mice, with 100 μL PBS containing DMSO (vehicle) or rapamycin, inhibits the mammalian target of rapamycin (mTOR) kinase, 30 min before injection with PBS or HK-*C. albicans*. Three days later, BMDMs were harvested and then stimulated with PBS or *C. perfringens*. Increased release of extracellular traps upon rechallenge with *C. perfringens* was observed from the BMDMs primed with HK-*C. albicans* rather than non-primed BMDM (Figure 5(a)). Inhibition of macrophages with rapamycin prior to priming significantly reduced the trained memory response of ETs (Figure 5(b)). In the present study, we indicate that the mTOR activation is critical for the memory response of ETs.

Data are shown as the mean ± SEM. n = 3 per group. Data are from three independent experiments. Statistical significance is indicated by *p < 0.05, **p < 0.01.

## Discussion

In this study, we explored the memory response of extracellular traps in a trained immunity model. Pretreatment with heat-killed *C. albicans* boosted the responses of macrophages against a subsequent *C. perfringens* infection, showed increased release of extracellular trap and cytokine. These effects in mice correspond to the described trained immunity characteristics and were recapitulated in chicken monocytes. Based on this, we propose that extracellular trap can be trained as a memory response. This notion was first described for exposure to heat-killed *Candida albicans*, which can protect the host against reinfection, enhance extracellular killing, and increase extracellular trap formation. *C. perfringens* is a zoonotic bacterium that infects humans, domestic animals, and poultry worldwide. In poultry, *C. perfringens* infection leads to necrotic enteritis [30], causing huge economic losses [31]. In our study, the trained memory of extracellular traps promotes *C. perfringens* clearance. It may enhance our understanding of host–pathogen interaction in bacterial infection. Such a prevention has the potential to reduce the harm of pathogen virulence.

Intraperitoneal injection of mice with heat-killed *C. albicans* resulted in typical features of trained immunity. Trained immunity enhances nonspecific antimicrobial responses against secondary infections and leads to enhanced pro-inflammatory cytokines production and phagocytosis in innate immune cells [32]. Consistent with previous research, in this study, primed BMDMs stimulated with a second stimulatory signal ex vivo showed a relatively higher TNF-α production. Besides, the extracellular bacteria of HK-*C. albicans*-primed BMDM were significantly decreased. Previous studies have shown that ETs are used as an immune response to kill invading bacteria [33], which were composed primarily of chromatin DNA, histones, and granule proteins [34]. In our study, we observed a significantly higher release of DNA and higher expression of citrulline histone 3. As we knew, activation of peptidylarginine deiminase 4 catalyzes the post-translational modification of arginine to citrulline and drives the formation of extracellular traps. As we expected, the protein expression of PAD4 was upregulated. All of the above studies clearly indicate a memory response of ETs formation, which enhances the innate immune response against *C. perfringens*. Initially, extracellular trap is regarded as an effective method for trapping and killing invasive bacteria. Subsequently, numerous evidence suggests that NET participates in the pathogenesis of autoimmune and inflammatory disorders [35]. In addition to classic neutrophil extracellular traps, other leukocytes are confirmed to release extracellular trap structures [36–38]. The macrophage and monocyte extracellular traps participate in host defense and disease states. Insufficient ET formation results in ineffective antimicrobial defense; however, excessive ET formation disrupts homeostasis and leads to various diseases [39,40]. This indicates that ET is a double-edged sword of innate immunity. Although, in our present studies, the memory response of extracellular traps contributes to extracellular bacteria killing, once long-term excessive extracellular traps release will lead to various ETs-associated diseases and exacerbate inflammation. A review supports this hypothesis that the trained memory of ETs increases the risk of developing autoimmune diseases. The review points out that low-grade endotoxemia (LGE) might induce trained immunity with the neutrophils’ response to the exaggerated NET formation, leading to gingival ulcerations and bacteremia/endotoxemia [41]. Understanding the mechanisms that regulate the trained memory of ET might be potential drug targets for treating infections or autoimmune diseases.

Trained immunity is an evolutionarily conservative innate immune mechanism that exists in multiple vertebrate species including mammals [26], chickens [28], and fishes [42]. This study showed that exposure to *C. perfringens* increases the production of NO in primed PBMC compared to non-primed. Meanwhile, our data also showed an increased extracellular DNA release and an increased bacteria killing capacity in primed PBMCs. It suggests that the memory response of ETs has no differences between mice and chicken, which may be mediated by a conservative mechanism in multiple species.

Multiple sources of β glucan, including oat, mushrooms, and yeast, could induce trained immunity in macrophages [43,44]. Although β-glucan is correlated with the activation of the Dectin-1 receptor, Dectin-1 receptor activation did not perform as a sole predictor for β-glucan mediated trained immunity [44]. Similar to β-glucan, mannan derived from the yeast cell wall plays a pivotal role in activating the innate immunity [22]. In previous studies, priming with β-glucan enhanced the production of TNF-α and IL-6 instead of mannan derived from *C. albicans* [16]. In our study, priming with yeast-derived mannan enhanced the formation of extracellular traps, which is similar to HK-*C. albicans*. It is indicated that the memory response of ETs could be induced by mannan. This discrepancy may be explained by different mechanisms to activate memory response in cytokines and extracellular traps.

Compared with classical adaptive immune memory, innate immune memory is generally reversible and shorter-lived. The immunological phenotype of trained immunity lasts 3 months to 1 year [45]. In this study, we observed the beneficial effects of pre-treatment with HK-*C. albicans* at a short term, the time interval between the two stimulations was 3 days. Future questions will address whether the memory response of the extracellular traps is a long-term effect. This investigation will help us understand the duration of the protection provided by the ETs memory response.

In conclusion, we discovered an association between trained immunity and extracellular traps, which confirms the memory response of ETs in both mammals and poultry. It provides a novel method to detect trained immunity and approves extracellular DNA release to evaluate trained immunity. It also offers novel ideas for the prevention and treatment of diseases. Here, we verify that priming signals induce the memory response in extracellular traps, and that increased ETs release upon re-infection leading to the heightened killing ability of extracellular bacteria. Excessive ETs release will cause inflammation and tissue damage. Modulation of the memory response of ETs might be a promising approach to diminish autoimmune disease. However, the detailed mechanisms and the optimal duration remain to be further explored.

## Supplementary Material

Supplemental Material

## Data Availability

The authors confirm that the data supporting the findings of this study are available within the article and its supplementary materials.
