# Supplementary figures and images for: Extracellular trap can be trained as a memory response
Source: Virulence. 2022 Mar 7;13(1):471–82. doi: 10.1080/21505594.2022.2046950 (PMC8903778; doi:10.1080/21505594.2022.2046950)

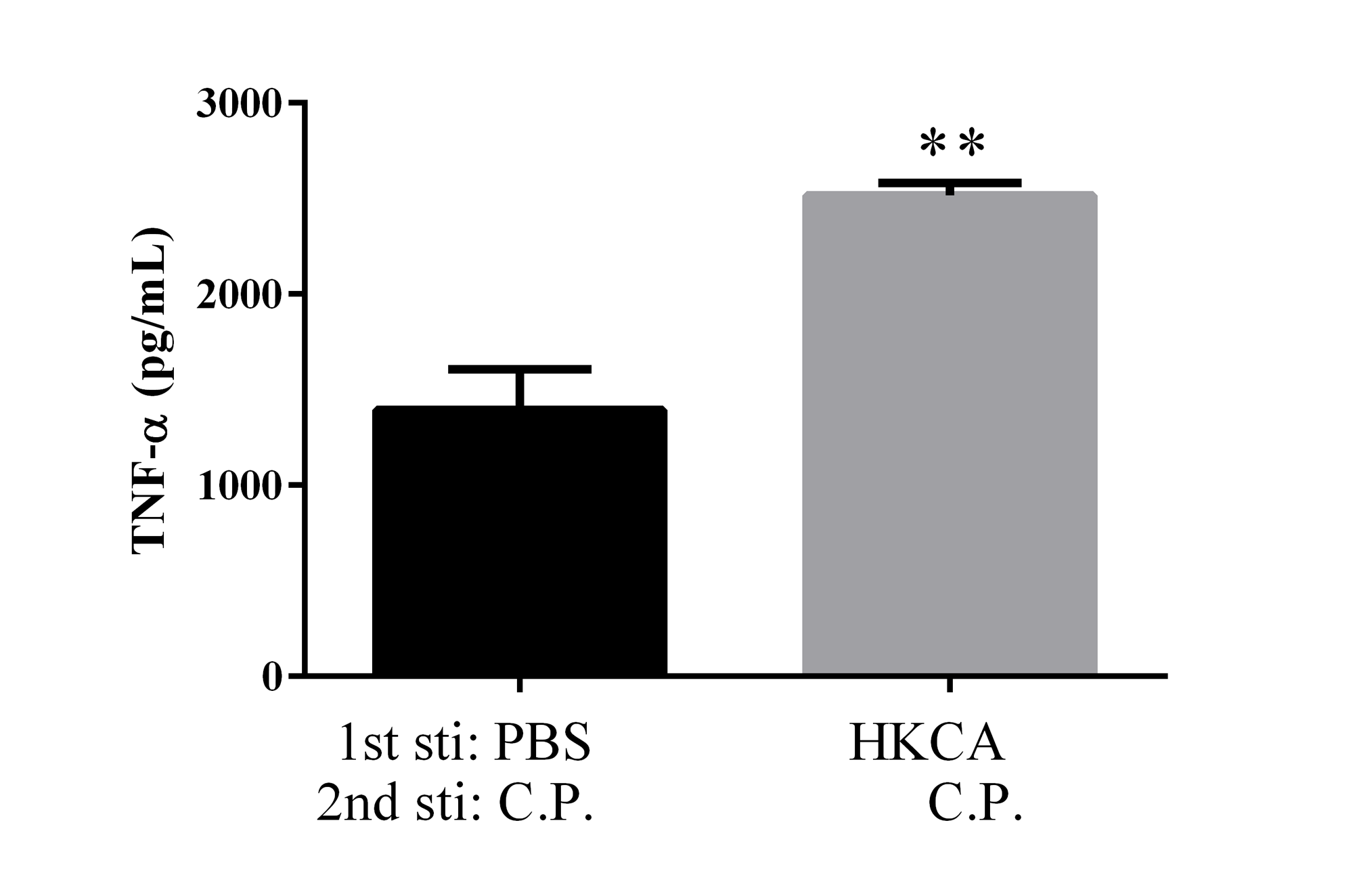

Supplement: Supplemental Material [file KVIR_A_2046950_SM5963.zip › Figure S1.tif]

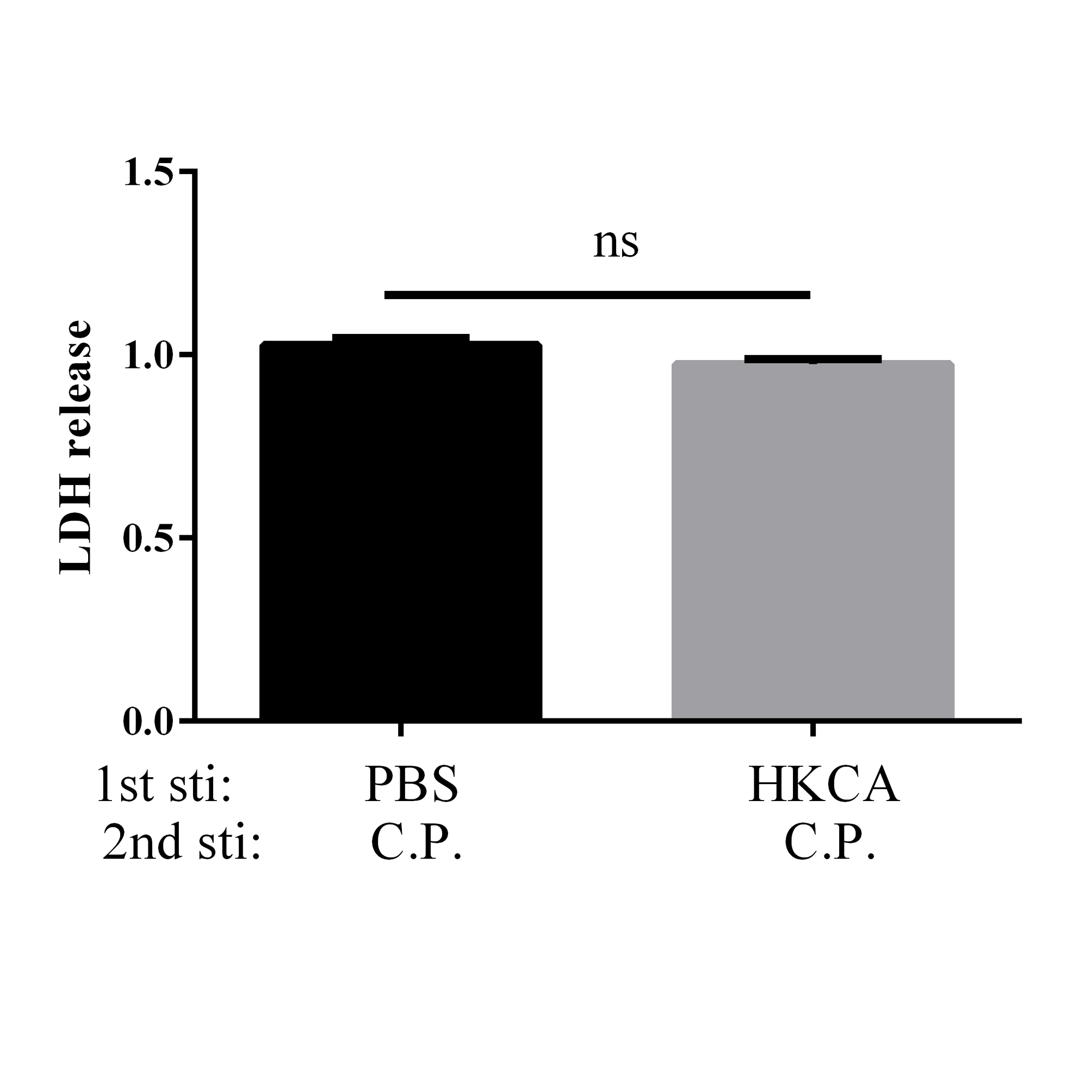

Supplement: Supplemental Material [file KVIR_A_2046950_SM5963.zip › Figuren S2.tif]
